# SARS-CoV-2 Aiming for the Heart: A Multicenter Italian Perspective About Cardiovascular Issues in COVID-19

**DOI:** 10.3389/fphys.2020.571367

**Published:** 2020-11-06

**Authors:** Matteo Briguglio, Mauro Porta, Francesca Zuffada, Alberto R. Bona, Tiziano Crespi, Fabio Pino, Paolo Perazzo, Marco Mazzocchi, Riccardo Giorgino, Giuseppe De Angelis, Alfonso Ielasi, Giuseppe De Blasio, Maurizio Turiel

**Affiliations:** ^1^IRCCS Orthopedic Institute Galeazzi, Scientific Direction, Milan, Italy; ^2^IRCCS Orthopedic Institute Galeazzi, Neurology Unit, Milan, Italy; ^3^ICCS Istituto Clinico Città Studi, Cardiology Unit, Milan, Italy; ^4^ICCS Istituto Clinico Città Studi, Neurosurgery Unit, Milan, Italy; ^5^IRCCS Orthopedic Institute Galeazzi, Intensive Care Unit, Milan, Italy; ^6^University of Milan, Residency Program in Orthopedics and Traumatology, Milan, Italy; ^7^ASST Rhodense, Cardiology Unit, Rho, Italy; ^8^Istituto Clinico Sant’Ambrogio, Cardiology Unit, Milan, Italy; ^9^IRCCS Orthopedic Institute Galeazzi, Cardiology Unit, Milan, Italy

**Keywords:** cardiovascular system, coronavirus, SARS-CoV-2, COVID-19, infections, virulence, host-pathogen interactions, quality of health care

## Abstract

The rapid spread of severe acute respiratory syndrome coronavirus 2 (SARS-CoV-2) and the high fatality rate of coronavirus disease 2019 (COVID-19) have been putting a strain on the world since December 2019. Infected individuals exhibit unpredictable symptoms that tend to worsen if age is advanced, a state of malnutrition persists, or if cardiovascular comorbidities are present. Once transmitted, the virus affects the lungs and in predisposed individuals can elicit a sequela of fatal cardiovascular consequences. We aim to present the pathophysiology of COVID-19, emphasizing the major cellular and clinical manifestations from a cardiological perspective. As a roaming viral particle or more likely *via* the Trojan horse route, SARS-CoV-2 can access different parts of the body. Cardiovascular features of COVID-19 can count myocardial injuries, vasculitis-like syndromes, and atherothrombotic manifestations. Deviations in the normal electrocardiogram pattern could hide pericardial effusion or cardiac inflammation, and dispersed microthrombi can cause ischemic damages, stroke, or even medullary reflex dysfunctions. Tailored treatment for reduced ejection fraction, arrhythmias, coronary syndromes, macrothrombosis and microthrombosis, and autonomic dysfunctions is mandatory. Confidently, evidence-based therapies for this multifaceted nevertheless purely cardiological COVID-19 will emerge after the global assessment of different approaches.

## The Journey of SARS-CoV-2

The little understanding of the natural diversity of the severe acute respiratory syndrome-related coronaviruses (SARS-CoVs) restricts the opportunities to control their zoonotic spillovers (Coronaviridae Study Group of the International Committee on Taxonomy of Viruses, 2020). Humans are therefore increasingly affected by outbreaks that put millions of people at risk. After the plagues of severe acute respiratory syndrome coronavirus 1 (SARS-CoV-1) in 2003 and of Middle East respiratory syndrome-related coronavirus (MERS-CoV) in 2012, a familial coronavirus (SARS-CoV-2) was discovered after the first documented virus-related pneumonia in China at the end of December 2019. This new strain is primarily transmitted through respiratory droplets and is able to survive in the airway mucosa despite the presence of cleaning epithelial cells, protective lymphoid tissues, and immunocompetent nerve endings (Briguglio et al., 2020a). The optimized genomic feature to bind to the angiotensin-converting enzyme 2 (ACE2), which derives from either millions of random natural mutations during unnoticed human-to-human transmission (Xu et al., 2020a) or artificial laboratory manipulations (Andersen et al., 2020), is the major determinant for the highest viral replication (Hoffmann et al., 2020) and for the consequent respiratory (Harapan et al., 2020) and cardiovascular implications (Wu et al., 2020b). After acquiring a sufficiently high viral load in the upper cavity (Zou et al., 2020), SARS-CoV-2 infects the goblet and ciliated cells in charge of sputum expectoration (Sungnak et al., 2020). The diffusion through the mucous layer allows the ease of infection of alveolar epithelial type II cells and systemic organs that express ACE2 (Briguglio et al., 2020a). The resulting illness, named coronavirus disease 2019 (COVID-19), is multifaceted and unpredictable and can manifest with early smell disorders in over 80% of cases or result in the most severe conditions like sepsis-like shock or respiratory failure in 14% of cases (Remy et al., 2020; Wu and McGoogan, 2020). Globally, it has been observed that 1 in 16 patients has encountered fatal consequences (WHO situation report 132, May–June 2020), and several infected patients were old and malnourished (Briguglio et al., 2020d; Sattar et al., 2020). Importantly, epidemiological data have been shown that preexisting cardiovascular conditions could be another central virulence factor for disease progression. In addition, clinical findings showed that not a few numbers of COVID-19 patients encounter cardiac symptoms (Mehra et al., 2020). Since each structure and function of the cardiovascular system shows severe implications, it is crucial to discuss from a cardiological perspective the relationship between SARS-CoV-2 infection and the cardiovascular system in order to shed some light on the mechanisms that can lead to cardiac symptoms or fatal consequences in COVID-19 patients.

## Virus-Associated Damage, Phases of Disease, and Patient Classification

It is necessary to differentiate the types of SARS-CoV-2-associated damages, the various stages of the disease, and the classification of infected patients. The virus-associated damage is of two types:

- Type I damage (i.e., cytotoxicity), which is directly associated with the infiltration of the virus in those cells expressing ACE2 (pneumocytes, endothelial cells, cardiomyocytes, neuronal cells). This may lead to acute injuries in the lungs, the vasculature, the myocardium, and the brain (Kabbani and Olds, 2020; Mason, 2020).
- Type II damage, which occurs during the disease progression. It derives from hypoxemia, inflammation, and microthrombosis. In particular, pneumonia and acute respiratory distress syndrome are likely to lead to a mismatch between oxygen supply and demand (hypoxic damage). Moreover, the late increase in circulating cytokines is known to cause nonischemic multiple organ injuries (e.g., stress-cardiomyopathy, myocarditis, vasculitis-like syndromes), and the systemic inflammation or catecholamine rush are associated with plaque rupture or blood hypercoagulability (i.e., thrombi-derived ischemic damage; Basu-Ray et al., 2020; Matsushita et al., 2020; Xiong et al., 2020; Zheng et al., 2020).

Considering the disease progression, three distinct phases have been recognized, covering the early infection mechanisms, the body’s response to the viral proliferation, and the late systemic phase of the illness.

- The incubation/proliferative phase: mild-to-moderate symptoms with fever, dry cough, headache, pharyngodynia, asthenia. This phase is biochemically characterized by mild lymphopenia and variations in some coagulation parameters, such as the D-dimer, thrombocytes, and international normalized ratio (INR). Lactate dehydrogenase as well as inflammatory markers like C-reactive protein and interleukin-6 may increase (Shi et al., 2020). Therapies to boost the immune response are certainly worth considering since early B lymphocyte reduction affects antibody production (Siddiqi and Mehra, 2020). This phase usually lasts a few days (Briguglio et al., 2020a).
- The respiratory phase: moderate-to-severe respiratory symptoms like shortness of breath and measurable hypoxemia. If a dysfunctional immune system was present, SARS-CoV-2 could proliferate quickly and lead to massive impairments of infiltrated tissues. This phase is characterized by increasing circulating levels of cytokines and chemokines, such as tumor necrosis factor-α, interleukins, interferon-γ, and chemoattractant proteins (Rokni et al., 2020). As long as the disease worsens, structural consequences include multiple patchy shadows in the lungs in mildly affected individuals or pleural fluid in the most severe cases (Yang et al., 2020). This phase normally starts to aggravate around 7–14 days after onset (Briguglio et al., 2020a).
- The systemic phase: moderate-to-severe systemic implications comprising acute distress respiratory syndrome, heart failure, and multisystem organ dysfunction. Troponin I and brain natriuretic peptide may be elevated in infected patients with cardiac involvement. The coagulopathy manifests with increased D-dimer and other fibrin degradation products, low platelet counts, and increased INR and prothrombin time (Lippi et al., 2020; Thachil et al., 2020). Severe lymphopenia, kidney injury, as well as elevated liver enzymes and cytokines may be found (Shi et al., 2020). Of note, lymphocyte attachment to the activated endothelium, together with their systemic redistribution and apoptosis, is supposed to be at the basis of low lymphocyte counts (Rokni et al., 2020). This phase might be conversely replaced by a recovery phase if the virus is effectively suppressed (Lin et al., 2020).

On the clinical bases, patients can be classified according to respiratory autonomy (Briguglio et al., 2020a):

- Level 0: asymptomatic, mostly home living.
- Level 1: mild symptoms, pharyngodynia, dry cough, mild fever; these individuals should not be hospitalized.
- Level 2: moderate symptoms, high fever, persistent cough, asthenia, dyspnea; these patients might require noninvasive oxygen therapy.
- Level 3: severe symptoms; these patients require invasive oxygen therapy and intensive care support. These patients were reported to meet the diagnostic criteria for sepsis, with the impaired liver, kidney, and lung functions presenting concomitantly with cold extremities, weak peripheral pulses, shock, and severe metabolic acidosis (Li et al., 2020b).

## From Lungs to Myocardium Injuries

The cardiovascular sequelae start with the viral binding to ACE2 in the lower airways, causing type I damage in pneumocytes (Leung et al., 2020). The altered diffusion of oxygen across the injured alveolar membrane is likely to ground hypoxic conditions that prevent proper tissue oxygenation. Locally, SARS-CoV-2 particles activate alveolar macrophages and T cells (Shi et al., 2020). The subsequent inflammation is known to stimulate hyaline membrane formation, wall thickening, and infiltration of circulating monocytes that differentiate into macrophages or fibroblast-like cells called fibrocytes that eventually favor fibrotic processes in the parenchyma (Pilling and Gomer, 2012). During the worsening of the respiratory phase, the overactive immunological response in the lungs alters the integrity of epithelial-endothelial barriers, with plasma components exuding in the alveolar cavity together with chemotactic monocytes and neutrophils (Li et al., 2013). In level 2 and level 3 patients, a cytokine storm might arise, being the main root for growing a worsening life-threatening systemic phase (Xu et al., 2020b). The recruitment of different leukocyte populations in the lungs could expose these cells to viral infiltration, ending up becoming Trojan horses (i.e., vectors for SARS-CoV-2, recall of the mythical subterfuge to enter the city of Troy). This mechanism was in fact shown for the familial predecessor SARS-COV-1 (Chen and Hsiao, 2004; Gu et al., 2005) and supposed for SARS-CoV-2 (Li et al., 2020b; Park, 2020), whose viral particles were found in blood samples and in the myocardium (Tavazzi et al., 2020; Wang et al., 2020b). If SARS-CoV-2 was able to infiltrate into the heart, it would be likely to elicit the secretion of cytokines from cardiac fibroblasts to subsequently increase the inflammatory milieu (van Nieuwenhoven and Turner, 2013) and to cause the recruitment of transendothelial monocytes (Lindner et al., 2014), neutrophils, and dendritic cells (Van der Borght and Lambrecht, 2018). Activated dendritic cells are known to trigger T cells (Eriksson et al., 2003), further promoting tissue damage. A plethora of immune cells, comprising macrophages and fibrocytes, may therefore populate these early myocardium lesions (Oudit et al., 2009; Pilling et al., 2009), each likely to have its own role in COVID-19-associated myocarditis and stress-cardiomyopathy (Xiong et al., 2020). Consequently, it would seem fair to assume that the myocardium of infected patients might be subjected not only to type I damage, as a consequence of direct myocardial cell injury, but also to type II damage mainly comprising the inflammation-derived grievance. Remarkably, even patients with mild respiratory symptoms can manifest early cardiovascular implications, such as acute myopericarditis (Inciardi et al., 2020), Takotsubo syndrome (Meyer et al., 2020), or acute myocardial infarction (Stefanini et al., 2020). Fulminant myocarditis was reported in level 2 patients (Hu et al., 2020; Zeng et al., 2020), and supraventricular tachycardia, decompensated heart failure, and cardiogenic shock were observed in aggravating level 3 patients (Fried et al., 2020). It is generally agreed that the lymphocytic count mirrors the nutritional status of the host (Briguglio et al., 2019), and it may be useful in predicting the patient’s reservoirs against the infection since these cells decline as long as COVID-19 worsens (Peteranderl and Herold, 2017; Chan et al., 2020). This attenuated immune potential of the host increases the susceptibility to disease complications, and the coupling of severe pneumonia with myocardial injury is likely to lead to progressive cardiorespiratory deterioration. Severe patients were in fact reported to be 13-fold more exposed to cardiovascular complications than non-severe, with an increased troponin I and low-density epicardial adipose tissue possibly reflecting the extent of the damage to the myocardium (Hui Hui et al., 2020; Li et al., 2020a), ultimately known to be associated with a worse prognosis (Clerkin et al., 2020).

## Endothelial Dysfunction and Atherothrombotic Manifestation

Endothelial dysfunction is a feature of COVID-19 that lingers from the proliferative to the systemic phase. If the viral load is high, probably boosted by an intense viral shedding in the blood flow (Chang et al., 2020), it is very likely that some particles directly affect the endothelium (Escher et al., 2020; Sardu et al., 2020). High levels of pro-inflammatory cytokines are associated with endothelial engrossment (Finkel et al., 1992; Cheng et al., 1999) that could progress to vasculitis-like syndromes in the vessels of the brain, the kidneys, or the gastrointestinal tract (Varga et al., 2020). In severe COVID-19 patients, the Kawasaki disease has been observed (Jones et al., 2020) together with cutaneous signs, such as the “COVID-19 toes” (Mazzotta et al., 2020) or the chilblain-like lesions (Papa et al., 2020). We can therefore assume that the endothelial dysfunctions in COVID-19 arise from both type I damage and the nonischemic type II damage. The dysfunctional endothelium elicits two events that are part of the “two-activation theory of the endothelium” (Chang, 2019): the release of inflammatory cytokines triggers the activation of inflammatory pathways, whereas the activation of the platelet and exocytosis of aberrant coagulation factors trigger the activation of microthrombotic pathways. Viruses are known to directly affect hemostasis with their ability to agglutinate platelets, cause hemolysis, and lead to the formation of procoagulant complexes with antibodies (McKay and Margaretten, 1967; van Gorp et al., 1999). This latter mechanism may be advocated for SARS-CoV-2 by recent computational modeling that showed the possibility of the virus to cause hemoglobin derangements (Liu and Li, 2020). If this were the case, then the incorporation of the virus into Trojan horses would be plausible since white cells are known to commonly engulf hemoglobin in various tissues (Briguglio et al., 2020c). Aberrant coagulation is the underlying mechanism for ischemic heart disease, stroke, and venous thromboembolism, but it has been observed also in severe influenza pneumonia and SARS-CoV-1 (Chong et al., 2004; Yang and Tang, 2016). Similarly, the development of coagulopathy appears to be a noxious complication in severe level 2 and level 3 patients (Tang et al., 2020b). Clots can be found in kidney dialysis catheters, cause strokes, or leave portions of lungs bloodless. Spleen atrophy, hilar lymph node necrosis, and hepatomegaly were also observed (Li et al., 2020b). Thrombus formation was associated with increased mortality (Zhou et al., 2020), with most of level 3 patients meeting the criteria for the disseminated intravascular coagulation (i.e., consumptive of both platelets and clotting factors; Lillicrap, 2020). Once thrombi formed in capillary beds, the remodeling processes would be associated with leukocyte polarization and late recruitment of macrophages that are in charge of cell clearance and blood flow restoration through fibrinolytic processes (Pober and Sessa, 2014). This cascade of events (Virchow’s triad) is nevertheless necessary for endothelial wall restoration (Mukhopadhyay et al., 2019). However, the immune derangements in COVID-19 are likely to alter the activation of both immune cells and the fibrinolytic system. For instance, neutrophil extracellular traps (NETs) are useful to entrap viruses in weblike structures, thus facilitating cleavage by macrophages. If neutrophils are abnormally activated, aggregated NETs and their associated antimicrobial factors may be key determinants in capillary destruction (Cicco et al., 2020), vessel obstruction (Leppkes et al., 2020), and lung injury (Wang et al., 2020a). Similarly, impaired activation of the fibrinolytic system activation can recirculate the material and thus increase the risk of distant thrombi-derived ischemic damages (i.e., disseminated intravascular microthrombosis). Although it is not known if a plaque rupture is as dangerous as the plaque before rupture (Schoenhagen et al., 2002), if circulating thrombi halt in the small coronary vessels, they can certainly contribute to myocardial injury (Hendren et al., 2020). Thromboembolic events can occur in the lungs of infected patients (Ai et al., 2020; Danzi et al., 2020), further impairing gas exchange. The pulmonary damage leads to poor perfusion in the coronary vessels, misbalance of oxygen supply/demand, reduced activity of the mitochondrial electron transport chain, acidosis, and oxidative damage from reactive oxygen species (ROS; Wu et al., 2020b), whose accumulation is also known to be elicited by the cytokine storm (Bhaskar et al., 2020). Importantly, tissue hypoxia is known to induce metabolic reprogramming in cardiomyocytes, thus being critical for the progression of numerous cardiovascular diseases (Abe et al., 2017, 2019).

## Electrical Dysregulation, Medullary Reflex Alteration, and Autonomic Dysfunction

Alike myocardial injuries, not all COVID-19 patients who manifest alterations in the cardiac electrophysiology, such as ST-segment or ST-T wave abnormalities, show concomitant chest tomographic opacities (Bangalore et al., 2020). It is therefore possible that in predisposed individuals, the cardiovascular system is affected before the respiratory system, with electrical dysregulations being caused by circulating levels of pro-inflammatory cytokines, stress hormones, electrolytic imbalances, or drug cardiotoxicity (Chung et al., 1990; Yokoyama et al., 1993; Hasan, 2013; Driggin et al., 2020), but SARS-CoV-2 might directly damage nerve fibers. The myocardium is innervated by sympathetic and vagal parasympathetic nerve fibers that intersect in local plexuses, ganglia, and pacemaker regions. The wide expression of ACE2 in nerve tissues and the neurotrophic nature of SARS-CoV-2 might render the cardiac nerve fibers a favorite prey (Briguglio et al., 2020a). Severe arrhythmias are nevertheless life-threatening conditions that may occur in over 30% of level 2 patients (Ferrari, 2020) and in higher rates in patients of level 3 (Huang et al., 2020). The prevalence and severity of electrocardiographic changes could reflect the progression of myocardial damage (Guo et al., 2020), but it is very likely that it is associated with disease progression. Defects of electrical impulses from the sinoatrial node to the ventricles might arise as drug-induced disorders, thereby requiring careful assessments before defining the pharmacological treatment of COVID-19 (Yogasundaram et al., 2014; Borba et al., 2020). Pulmonary stretch receptors, C-fibers in the alveolar wall, baroreceptors in the carotid sinuses, extra-carotid cardiopulmonary baroreceptors together with widespread metaboreceptors are critical for integrating breathing cycle, heart rate, and vascular resistance during ventilatory and arterial pressure changes (Sant’Ambrogio, 1982; Schelegle, 2003; Timmers et al., 2003; Kougias et al., 2010; Anand et al., 2014). Type II damages are likely to disrupt these nervous components, in turn compromising the responsiveness to local stimuli, the impulse activity in afferent glossopharyngeal and vagal fibers, and the reflexive outflow (Burki and Lee, 2010; van Gestel and Steier, 2010). The central processing would therefore receive vitiated information from the periphery, which grounds the lack of adaptation of intrapulmonary vessels of COVID-19 patients (Chu et al., 2020), with the outputs being equally artificial. For instance, it has been suggested that the state of “silent hypoxemia” (i.e., depressed dyspnea response) that was observed in a large number of COVID-19 patients could be associated with defects in the carotid body, which is known to express ACE2 (Tobin et al., 2020). The consequent poor regulation of blood displacement in the microcirculation to the lungs and the brain may therefore mirror a vitiated baroreceptor reflex and hemodynamics, as was indeed observed in a COVID-19 patient (Ribas et al., 2020). Importantly, countless cardiovascular implications have been associated with the frequent renal involvement that was observed in level 2 and level 3 patients (Ronco et al., 2020). It is reasonable to believe that kidneys are subjected to both viral infiltration and several types of type II damages (Larsen et al., 2020; Su et al., 2020). Local polarization and subsequent activation of white blood cells easily disrupt the renin-angiotensin-aldosterone system (RAAS; Strutz and Zeisberg, 2006; Chen et al., 2016; Granot et al., 2017), in turn affecting the sympathetic noradrenergic and parasympathetic cholinergic neurotransmission (Miller and Arnold, 2019). Nevertheless, this intense extended autonomic system (EAS) activation was suggested to account for the multiple organ involvement of COVID-19 (Goldstein, 2020). Other than arrhythmias, level 3 patients were reported to be subjected to more frequent vasopressor support (Goyal et al., 2020). Some of these patients showed clinical involvement of the brainstem, especially of the respiratory center (Manganelli et al., 2020), which can imply a type I damage of SARS-Cov-2 *via* cerebrospinal fluid diffusion (Sun and Guan, 2020) or vagus nerve retrograde transport (Tassorelli et al., 2020). The autonomic center at the level of the lower medulla expresses ACE2 (Xia and Lazartigues, 2008), and it was shown to be highly infected by familial predecessors (Netland et al., 2008; Li et al., 2016). Non-epileptic seizures due to autonomic dysfunction were indeed reported in a COVID-19 patient (Logmin et al., 2020). The systemic inflammation, ischemic thrombotic/cardio-embolic injuries, or vasculitis at the level of capillary beds beneath the ependyma of the ventricle may similarly affect brainstem functions (Benghanem et al., 2020; Mirza and Das, 2020), being all hallmarks of the systemic phase of COVID-19. Endothelial damages in these critical areas are likely to affect afferent inputs from peripheral nerves, with subsequent lack of proper buffering of blood pressure fluctuations from the nucleus of the solitary tract (Cutsforth-Gregory and Benarroch, 2017). Clinically, the involvement of this medullary nucleus or of the dorsal motor nucleus of the vagus nerve might evoke nausea and vomiting frequently observed in COVID-19 patients (Goldstein, 2020). Electrical evaluation of both heart and brain activities, echocardiography, invasive hemodynamic monitoring, and serum brain natriuretic peptide can help clarify the cardiogenic component (Yufu et al., 2006; Mazeraud et al., 2016). Notably, any infection of central nervous tissues is accompanied by massive infiltration of leukocytes, such as dendritic cells from the perivascular region (Ludewig et al., 2016), that could serve as Trojan horses, further contributing to local affections.

## Preexisting Cardiovascular Conditions as Virulence Factor: Implications for Disease Onset and Progression

Although it is not possible to state whether the cardiovascular implications observed in COVID-19 derive from previous conditions or depend solely on the coronavirus-associated damages, it is reasonable to assume a causal link. From a molecular point of view, the upregulation of ACE2 in some cardiovascular diseases, such as ischemic heart disease or diabetes mellitus (Zisman et al., 2003), may certainly expose the sick individuals who contract the coronavirus to poorer prognosis (Wu et al., 2020b). The subsequent binding and downregulation of ACE2 expression by SARS-CoV-2 further prevent the conversion of angiotensin II, thus worsening pulmonary and cardiovascular outcomes (Datta et al., 2020). Accordingly, a higher ACE/ACE2 ratio might be a predisposing cause of worse outcomes in COVID-19, having angiotensin II dire vasoconstriction and pro-oxidant and pro-inflammatory effects in contrast to angiotensin (1–7) that is a vasodilator, antioxidant, and anti-inflammatory (Pagliaro and Penna, 2020). Clinically, it has been proposed that the more disturbed was the hemodynamic homeostasis prior to SARS-CoV-2 infection, the more severe could be the symptoms during COVID-19 and the higher would be the risk of long-term cardiovascular consequences (Zheng et al., 2020). Concerning the Italian cohort of patients, 1 in 3 had preexisting ischemic cardiomyopathy or diabetes mellitus, 1 in 4 already suffered from atrial fibrillation, and 1 in 10 had a history of stroke (Onder et al., 2020). The preexisting myocardial metabolic imbalances or atherosclerotic lesion might have played a major role in myocardial oxygen imbalances and plaque instabilities upon the advent of the systemic phase of COVID-19 (Bonow et al., 2020). Numerous mechanical (e.g., repetitive deformations derived from the cardiac cycle) and biological forces (e.g., inflammation) are known to undermine the stability of subclinical plaques (Arroyo and Lee, 1999; Yao et al., 2019), and they all occur during infections (Madjid et al., 2007; Campbell and Rosenfeld, 2015). In addition, a preexisting poor cardiac functional reserve is more likely to lead to a sudden cardiac insufficiency in patients with COVID-19, giving also the drug-related heart damage deriving from COVID-19 treatment (Wu et al., 2020a; Zheng et al., 2020). In the past, patients with comorbid cardiovascular diseases, such as coronary artery disease or heart failure, have been already recognized to be at higher risk of contagion and exacerbation of symptoms during viral respiratory infections (Nguyen et al., 2016). Furthermore, long-term damage to the cardiovascular system has been documented in hospitalized patients recovering from pneumonia (Corrales-Medina et al., 2015), thus highlighting the cardiorespiratory deteriorations of COVID-19. It is therefore reasonable to say that any previous hypoxic/vascular condition, cardiac inflammation, or autonomic dysfunction has to be recognized as a risk factor for COVID-19 onset and cardiovascular disease progression in any individual infected with SARS-CoV-2. Notably, the highest case/fatality ratio in older adults might be due to the increasing prevalence of frailty and comorbid cardiovascular diseases in advanced age (Briguglio et al., 2020b; Moccia et al., 2020), which is known to be associated with increased ACE/ACE2 ratio (Wang et al., 2016). While it is still controversial whether RAAS inhibitors are to be administered to COVID-19 patients (Shibata et al., 2020), it is certain that the therapy with ACE inhibitors and angiotensin receptor blockers (ARBs) should definitely not be discontinued in patients with preexisting cardiovascular diseases (de Abajo et al., 2020; Vaduganathan et al., 2020).

## Conclusive Remarks

**Figure 1:**
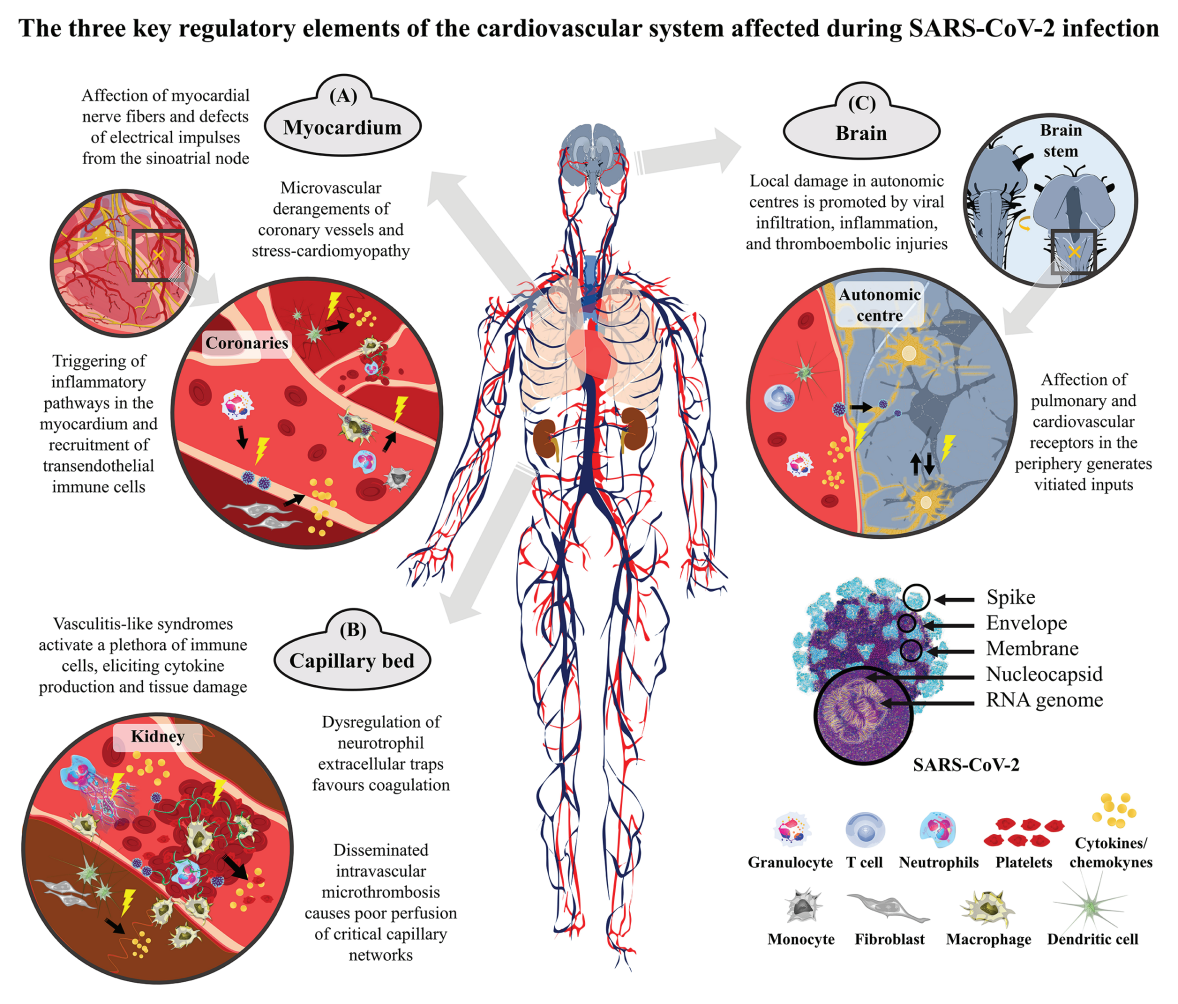
Representation of the cardiovascular derangements of coronavirus disease 2019 (COVID-19) that are due to either direct cellular damage of severe acute respiratory syndrome coronavirus 2 (SARS-CoV-2) or indirect consequences of the exaggerated host’s response. The severe acute respiratory syndrome coronavirus of 2019 (SARS-CoV-2) is a single-strand positive-sense RNA virus that spreads between humans mainly through the inhalation of respiratory droplets. Upon the collapse of the alveoli in the lungs, the virus can enter the bloodstream and distribute to systemic districts by cardiac pumping. It is likely that the transport in the blood is not as such, but carried by different types of white blood cells, such as T cells, granulocytes, and macrophages, which can therefore serve as vehicles. This Trojan route can guarantee the infiltration of the virus into normally inaccessible body districts. The systemic spread of the coronavirus elicits an exaggerated immune response in the most severe cases that strokes with hypoxic conditions. **(A)** In the myocardium, the excessive activation of the endothelial system upon viral damage and the enhanced inflammatory cell infiltration alter the coronary perfusion and the cardiac rhythm. Circulating monocytes and neutrophils infiltrate in the heart wall and parenchyma, with resident dendritic cells contributing to cytokine production and inflammatory/pro-fibrotic environment. **(B)** In the blood, the hyperactivation of both inflammatory and microthrombotic pathways leads to endothelium engrossment and coagulopathy. The activated vascular endothelium is targeted by neutrophils and monocytes, with thrombosis or bleedings being likely to occur because of the inflammation-derived imbalance between platelets, hypercoagulability, and altered fibrinolysis (fibrin in green). Thrombus-associated white blood cells produce inflammatory cytokines and proteases that contribute to local remodeling and fibroblast activation. Atherothombotic manifestations may also be promoted by dysregulation of neutrophil extracellular traps (NETs). **(C)** The heart is innervated by vagal postganglionic fibers from the cardio-inhibitory center and by the cardiac postganglionic fibers from the spinal cord arising from the cardio-acceleratory center of the medulla. Sympatho-inhibitory and cardio-inhibitory baroreflexes together with arterial metaboreflexes encompass inputs to neurons located in the dorsolateral nucleus of the solitary tract that integrate the vasomotor tone and the automatism of the sinus node. Damages to these reflexes disturb these central pathways and ultimately disrupt the heart beat nuclei, eventually leading to irrepressible dysautonomia.

COVID-19 is a multifaceted illness that comprises several implications of cardiological nature, including hypoxemia, sustained activation of the endothelium, nonischemic injuries, leukocyte polarization, thrombi-derived ischemic damages, dysrhythmias, and autonomic dysfunctions (Figure 1). Given these considerations, it is reasonable to conclude that the more severe autonomic dysfunctions of critically ill patients, the more complex would be the preservation of hemodynamic balances, thereby increasing the likelihood of fatal cardiovascular consequences in COVID-19 or chronic cardiovascular damages in those who survive. In these patients, long-term remote electrophysiological monitoring might be useful to provide care as necessary after discharge (Lakkireddy et al., 2020). Understanding these pathophysiological mechanisms in COVID-19 is crucial to promptly triage early risk factors, tailor treatment according to the patient’s severity and risk-benefit balance, and integrate evidence-based therapies depending on the disease phase (Carter et al., 2020; Mycroft-West et al., 2020). Drugs for COVID-19 have not been available yet (Kalil, 2020), but immunotherapies, extracorporeal membrane oxygenation, and low-molecular-weight heparin are being tested for effectiveness (Paranjpe et al., 2020; Perazzo et al., 2020; Ramanathan et al., 2020; Spyropoulos et al., 2020; Tang et al., 2020a; Thachil, 2020). In the meantime, cardiologists should stay up-to-date on recent and ongoing discoveries regarding COVID-19 and take a prominent role in the research studies or multidisciplinary teams.

## Data Availability Statement

The original contributions presented in the study are included in the article/supplementary material, further inquiries can be directed to the corresponding author.

## Author Contributions

MB formulated the hypothesis and wrote the first draft of the manuscript. MP, FZ, AB, TC, FP, PP, MM, RG, GA, AI, GB, and MT revised the first draft and contributed to manuscript sections. All authors contributed to manuscript revision and read and approved the submitted version.

### Conflict of Interest

The authors declare that the research was conducted in the absence of any commercial or financial relationships that could be construed as a potential conflict of interest.
